# Oligoarginine peptide structure and its effect on cell penetration in ocular drug delivery

**DOI:** 10.1016/j.heliyon.2024.e35109

**Published:** 2024-07-24

**Authors:** Stefana Duca, Naa Dei Nikoi, Madeline Berrow, Lois Barber, Louise N. Slope, Anna F.A. Peacock, Felicity de Cogan

**Affiliations:** a School of Pharmacy, University of Nottingham, Nottingham, UK; b Institute of Microbiology and Infection, University of Birmingham, Birmingham, UK; c School of Chemistry, University of Birmingham, Birmingham, UK

**Keywords:** Cell-penetrating peptides, Oligoarginine, Ocular drug delivery, Antibacterial

## Abstract

Oligoarginine cell-penetrating peptides (CPPs) are short peptides that can enhance drug delivery into cells and are of particular interest in ocular topical formulations for age-related macular degeneration (AMD) treatments. The length and structural characteristics of these peptides are considered crucial for drug delivery. This study investigates how oligoarginine length (R_n_) affects their penetration mechanism, drug delivery capabilities, and antimicrobial properties, providing insights into their potential roles in AMD treatment delivery. In this study, oligoarginine peptides showed limited pore-forming abilities in a carboxyfluorescein-containing liposomal model, with R_9_ being the only oligoarginine length recording a significant pore-formation level. Their antibacterial efficacy depended on both the CPP length and bacterial class, with longer peptides exhibiting stronger antibacterial effects. Importantly, oligoarginine was found nontoxic to relevant mammalian cells for ocular delivery. The membrane translocation abilities of oligoarginine were consistent regardless of cargo presence. Additionally, cargo delivery by oligoarginine across *in vitro* cellular models for ocular delivery was dependent on peptide length and cell type, with longer chains being more effective at cargo uptake in a corneal epithelium cell line, and with shorter chains proving more effective for cargo delivery in a retinal epithelium cell line. This proposes that the chain length of oligoarginine could be used as a strategic tool in the formulation process to selectively target distinct regions of the eye. Overall, this study expands our understanding of how oligoarginine CPPs can be applied as penetration enhancers to improve the delivery of therapeutics in an ocular topical formulation within the clinical context of AMD.

## Highlights

• The relationship between oligoarginine chain length and delivery efficacy was studied.
• Longer oligoarginine peptides show stronger antimicrobial effects.
• Pore formation is not the principal mechanism of penetration of oligoarginine.
• Oligoarginine was non-toxic in an *in vitro* cellular model relevant to ocular delivery.
• Oligoarginine chain length may permit the selective targeting of distinct eye regions.

## 1 Introduction

Cell-penetrating peptides (CPPs) are short peptides that can deliver membrane-impermeable molecules inside the cytosol due to their ability to translocate through cell membranes to access intracellular structures [1]. The use of CPPs as a tool to access the cell by acting as penetration enhancers for therapeutics and biological molecules of interest is well established [1]. CPPs have the advantage of having a low-cost, simple manufacturing process and being biocompatible and readily processed by the body. In a clinical setting, CPPs can be formulated for topical administration to enhance the ocular absorption of age-related macular degeneration (AMD) therapies, such as protein-based therapies (e.g., anti-vascular endothelial growth factor agents) and small peptide therapies (e.g., the novel therapeutic candidate Apolipoprotein A-I mimetic peptide 4F, ApoAI) [2,3]. This approach can help prevent serious side effects associated with the current AMD drug delivery platforms (i.e., intravitreal injections) and improve patient compliance [[4], [5], [6], [7]]. There are promising studies that have demonstrated the ability of CPPs to successfully deliver AMD agents to the posterior region of the eye both *in vitro* and *in vivo* whilst preserving drug action [8,9]. The most common type of CPP is a cationic peptide. As a class, they show excellent cellular intake across a wide range of cell types, with oligoarginines being the most utilised type for topical drug delivery [10].

Oligoarginines are polypeptides composed solely of arginine residues (R_n_) and possess a large positive charge that enables them to pass the cell membrane efficiently. Oligoarginine CPPs have previously been employed to deliver a range of molecules [9,11,12]. For instance, they have been utilised to form protein–protein complexes with insulin, thereby facilitating its delivery across the gut epithelium both *in vitro* and *in vivo* [11]. Similarly, the topical anti-inflammatory drug cyclosporine A, covalently linked to oligoarginines, demonstrated enhanced penetration through the outermost layer of the skin to access the underlying epidermis [12]. de Cogan et al. (2017) demonstrated the potential of oligoarginines as penetration enhancers for ocular delivery, as they successfully internalised anti-VEGF agents in an *in vivo* rat retinal model [9]. Furthermore, additional studies showed that oligoarginine peptides can form stable conjugates with anti-VEGF therapeutics like bevacizumab, further supporting their suitability as a novel form of AMD drug delivery technology [13]. While the ability of oligoarginine peptides to internalise therapeutic cargo into cells due to their high positive charge has been established, the exact mechanism by which they enter the cells remains controversial due to conflicting evidence in the literature [14,15].

Several studies have suggested that oligoarginine can be internalised via various mechanisms, such as absorption-mediated endocytosis and passive translocation via pore formation [14,15]. However, no definitive evidence establishes the precise mechanism behind their cell delivery abilities. The guanidine head group, a potentially crucial structure required for peptide uptake into cells, has been found to directly influence the translocation of oligoarginine within cells, with peptide length playing a key role in this process [10,16]. As such, it is believed that the length and charge of oligoarginine CPPs are critical factors in their drug delivery abilities. Studies have been conducted to evaluate the optimal oligoarginine length for efficient cellular uptake [10,16]. Some reports indicate that a minimum of six arginine residues (R_6_) is required for effective internalisation. In contrast, others propose that oligoarginine lengths of R_6_–R_8_ present optimal cargo uptake compared to longer variants, and still, other sources suggest that longer oligoarginines (e.g., R_10_ and R_12_) can achieve significant internalisation at lower concentrations than shorter oligoarginines (e.g., R_7_ and R_9_) [10,16,17]. However, no study to date has been conducted to investigate the optimal length of oligoarginines required for efficient ocular drug delivery.

Furthermore, in addition to their role as penetration enhancers in AMD therapeutics, oligoarginine peptides could potentially serve as preservatives in topical formulations, as several studies indicate that these peptides possess antimicrobial activity [18,19]. It has been suggested that arginine-rich polypeptides can act as antimicrobial peptides (AMPs) by inducing pore formation in bacterial cells [20]. As oligoarginine length appears to be critical in their delivery mechanism and CPP characteristics, chain length may also play a key role in their antimicrobial abilities.

Therefore, this investigation aimed to evaluate the structure–activity relationship of oligoarginine peptides as a novel drug delivery system for AMD therapeutics in the form of an ocular topical formulation. To accomplish this, the impact of oligoarginine structure and length (R_n_) on its drug delivery and antimicrobial capabilities was studied. In this study, we have examined oligoarginine-mediated delivery of both large and small target molecules. Bovine serum albumin-Texas Red (BSA-TR) was used as a representative model for protein molecules, and ApoAI (molecular weight 2.31 kDa) as a model for small peptide molecules [3,21].

## 2 Materials and methodology

### 2.1 Materials

1X Gibco Dulbeco's modified Eagle's medium (DMEM), F-12 containing l-glutamine and 15 mM HEPES buffer, foetal bovine serum (FBS), penicillin-streptomycin, 0.25 % 1X trypsin-EDTA were obtained from Gibco, UK. Trypan Blue and Texas Red-tagged BSA (BSA-TR) (LOT# 2136779) were from Invitrogen, UK. Dipalmitoylphosphatidylcholine (DPPC) phospholipid was purchased from Avanti® Polar Lipids, USA. All other reagents, including phosphate buffered saline (PBS), amphotericin B solution (250 μg/mL), chloroform, diethyl ether, Triton X-100, carboxyfluorescein, AlamarBlue® solution, and Luria-Bertani (LB) broth were procured from Merck, UK. Oligoarginine peptides (R_3_, RRR; R_6_, RRRRRR; R_9_, RRRRRRRRR; R_12_, RRRRRRRRRRRR) were purchased from GenScript, UK, as untagged peptides (R_n_) and carboxyfluorescein tagged peptides (FAM-R_n_). Carboxyfluorescein-tagged Apolipoprotein A-I mimetic peptide 4F (FAM-ApoAI) was obtained from Alta Bioscience, UK.

### 2.2 Liposome preparation

Carboxyfluorescein-containing liposomes were synthesised by adapting a protocol from Jimah et al. (2017) [22]. The phospholipid used for liposome preparation was DPPC, and the buffer of choice was HEPES-NaCl (10 mM HEPES pH 7.4 combined with 150 mM NaCl and deionised water). Initially, 10 mg of DPPC was dissolved in 1 mL chloroform. A phospholipid film was created by solvent evaporation under a gentle stream of nitrogen gas. The lipid film was re-hydrated by adding 500 μL of 20 mM carboxyfluorescein solution substituted in HEPES-NaCl buffer and 500 μL diethyl ether using a Pasteur pipette. This was sonicated three times for 60-s intervals at 50 °C and vortexed for 15 s between each sonication. Using a rotary evaporator, the solvent was removed at 25 °C, 500 mbar, and 100 RPM. An Avanti Mini Extruder set was used to obtain uniform-sized liposomes. The extruder was primed in HEPES-NaCl buffer and pre-warmed to 50 °C. Finally, the liposome suspension was purified using a desalting column (Cytiva, US) according to the manufacturer's instructions, and a liposome stock solution was prepared in HEPES-NaCl buffer in a 1:100 ratio.

### 2.3 Liposome disruption assay

Untagged oligoarginine peptides (R_n_) were prepared in HEPES-NaCl buffer at a concentration of 200 mM. A volume of 2 μL of liposome stock was added per well with sufficient HEPES-NaCl buffer to give a total volume of 100 μL, and the fluorescence was read at an excitation/emission wavelength of 485/535 nm using a TECAN plate reader. Subsequently, 1 μL of the 200 mM untagged oligoarginine stock solution or HEPES-NaCl buffer (acting as a negative control) was added per well and incubated for 10 min at room temperature, and fluorescence was read at the described settings. To ensure complete liposome disruption, 0.1 % Triton X-100 was added to each well. Triton X-100 is known for its ability to disrupt phospholipid membranes and served as a positive control to validate the assay [22].

### 2.4 Minimum inhibitory concentration (MIC)

A broth microdilution assay was performed to determine the minimum inhibitory concentration (MIC) for the oligoarginine peptides. *Escherichia coli* (*E. coli*) ATCC25922 and *Staphylococcus aureus* (*S. aureus*) ATCC6538 were used as model organisms for Gram-negative and Gram-positive bacteria, respectively. Both strains were cultured in LB broth for ∼18 h overnight at 37 °C and shaking set to 180 RPM. The culture was diluted to obtain a 0.01 optical density (OD) bacterial suspension in LB. Using a 96-well plate, oligoarginine concentrations ranging from 1 mM to 0.001 mM were obtained, and each peptide concentration was tested against both bacterial strains. The bacteria were incubated overnight at 37 °C for 18 h, and the lowest concentration of the treatment with no visible bacterial growth was recorded as the MIC of the respective oligoarginine treatment.

### 2.5 Cell culture

The *in vitro* cellular models were chosen to represent barriers relevant to ocular drug delivery, human keratocyte cells as a component of the corneal epithelium, and the retinal pigment epithelium and blood-retina barrier as ARPE-19 cells [23,24]. Primary human keratocyte cells (P10872, Innoprot, Spain) were cultured in DMEM/F12 media supplemented with 10 % foetal bovine serum (FBS), 1 % penicillin-streptomycin, 0.1 % amphotericin B, and 10 ng/mL basic fibroblast growth factor (b-FGF). ARPE-19 cells, a gift from the University of Birmingham, were cultured in DMEM/F12 media supplemented with 10 % FBS, 1 % penicillin-streptomycin, and 0.1 % amphotericin B. Cells were harvested for passaging or seeding once a cell confluency of >80 % was reached.

### 2.6 Cell toxicity

Keratocyte and ARPE-19 cells were seeded into 96-well plates at a density of 5 × 10^3^ cells/well in 50 μL of their respective cell media at 37 °C and 5 % CO_2_. After 24 h, cell media was removed, and fresh media supplemented with oligoarginine peptides at 56 μM was added to each well. Following a 72-h incubation at 37 °C and 5 % CO_2_, cell viability was measured using AlamarBlue® solution (0.1 mg/mL). AlamarBlue® uses the fluorometric redox indicator resazurin (7-hydroxy-3H-phenoxazin-3-one 10-oxide), initially a blue-coloured non-fluorescent compound. Once internalised by viable cells, resazurin is reduced to the fluorescent resorufin (7-hydroxy-3H-phenoxazin-3-one) allowing measurement of cell viability [25]. A volume of 5 μL/well AlamarBlue® stock solution was added in 45 μL/well cell media for 2 h. Each well was measured at a fluorescence wavelength of 545/590 nm using a TECAN plate reader. Subsequently, a cell count was performed by washing the cell monolayer three times in PBS and by harvesting the cells from the 96-well plate using 20 μL/well 0.25 % Trypsin-EDTA. The cells were counted in a 1:1 ratio with Trypan Blue, using a Countess automated cell counter (Invitrogen, UK). Performing a full cell count alongside a metabolic rate assay provides an understanding of the compound's toxicity beyond metabolic activity, as it directly quantifies cell numbers. Each polypeptide length was tested in triplicate across three experimental repeats, and a no-peptide positive control was included in each experiment.

### 2.7 Cell monolayer assay

Keratocyte and ARPE-19 cells were seeded into 96-well plates at a density of 4 × 10^4^ cells/well in 100 μL of their respective cell media at 37 °C and 5 % CO_2_ for 24 h. Prior to performing the assay, cell media was discarded, and cells were washed three times in PBS. A pre-treatment reading with 100 μL PBS at excitation/emission wavelengths of 485/535 nm (FAM-tagged molecules), 570/630 nm (Texas Red-tagged BSA, BSA-TR) or both was measured to identify background fluorescence reading. Subsequently, 50 μL/well of the constituted formulation (FAM-R_n_ only formulation: 25 μM FAM-R_n_ in PBS; FAM-R_n_/BSA-TR formulation: 200 μM FAM-Rn with 250 μg/mL BSA-TR in PBS; R_n_/FAM-ApoAI formulation: 2.5 mM R_n_ with 200 μg/mL FAM-ApoAI in PBS) was applied to the cell monolayer and incubated for 60 s. The solution was removed, and the wells were washed five times with PBS. A volume of 100 μL/well of PBS was added, and the fluorescence was read at excitation/emission wavelengths of 485/535 nm and/or 570/630 nm. Matching control samples treated with PBS only or cargo only (at the same concentrations used in their corresponding experiments) were included where appropriate.

### 2.8 Calibration curves of oligoarginine peptides and BSA

**Fig. 1:**
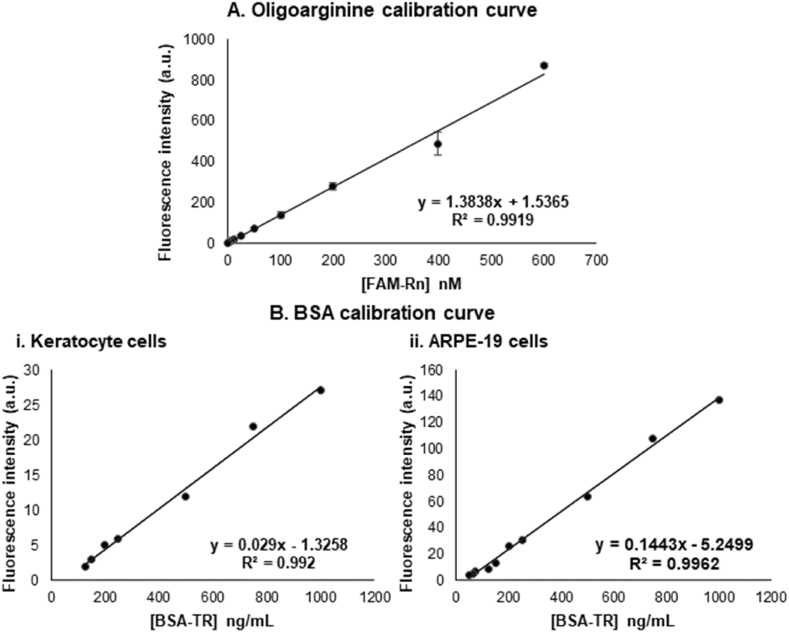
**Calibration curves for A. FAM-tagged oligoarginine peptides (FAM-Rn) and B. TexasRed-tagged BSA (BSA-TR) for B.i. keratocyte and B.ii. ARPE-19 cell monolayers.** Fluorescence intensity is measured in arbitrary units (a.u.). Data is presented as the mean ± SEM (n = 4) where applicable. The data was fitted using linear regression.

To estimate uptake of oligoarginine and BSA in cell monolayer experiments, calibration curves have been developed (Fig. 1). For FAM-tagged oligoarginines (FAM-R_n_), standards ranging from 7.8 to 600 nM were prepared for each peptide length. Due to consistent fluorescence readings across different peptide lengths, a single calibration curve was generated using the average fluorescence readings across all tested oligoarginines (Fig. 1A). Fluorescence was measured at excitation/emission wavelengths of 485/535 nm. Similarly, for TexasRed-tagged BSA (BSA-TR), standards with concentrations ranging from 50 to 1000 ng/mL were prepared, with fluorescence readings taken at excitation/emission wavelengths of 570/630 nm. Separate calibration curves were created for BSA-TR fluorescence in keratocyte (Fig. 1Bi) and ARPE-19 (Fig. 1Bii) cell monolayers to accommodate differences in measurement settings. All calibration curves were constructed in Microsoft Excel utilising the linear regression trendline function, ensuring an R^2^ value of ≥0.99 to validate the data fitting appropriately. The fitted equation (as seen in Fig. 1) was then used to estimate unknown concentrations of each treatment.

### 2.9 Statistical analysis

Statistical evaluation was performed using GraphPad Prism 10.0.2. All experiments included at least three experimental repeats with two, three, or more technical repeats, with matched control conditions. All datasets were tested for normality using the Shapiro-Wilks test. Where data failed the normality test (Shapiro-Wilk, p < 0.05), it was subject to log transformation to achieve a normal distribution. Differences in cell monolayer experiments were assessed with one-way ANOVA followed by Tukey's post-hoc test. In contrast, differences in liposome disruption assays and mammalian toxicity studies were assessed with one-way ANOVA followed by Dunnet's post-hoc test. Significance was taken at p ≤ 0.05. All data is presented as the mean ± standard error of the mean (SEM).

## 3 Results

### 3.1 CPP membrane-disruption abilities

**Fig. 2:**
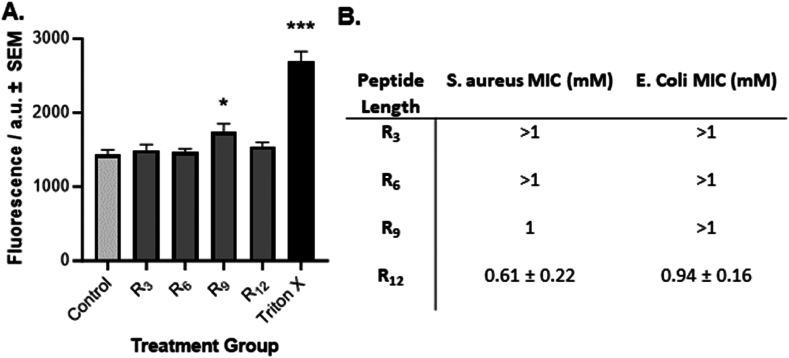
**Membrane penetration of oligoarginine CPPs.** A) Stimulated leakage of fluorescence from liposomes. Data shows the mean ± SEM (n = 9) of carboxyfluorescein fluorescence from liposomes following a 10-min incubation of the carboxyfluorescein-containing liposomes with oligoarginine peptides. Liposomes with no penetration agent added and 0.1 % Triton-X act as a negative and positive control, respectively. Fluorescence intensity is measured in arbitrary units (a.u.). Significance was taken at p ≤ 0.05 using One-way ANOVA and Dunnett's post-hoc test. *p < 0.05 ***p < 0.001 when compared to Control. B) Antimicrobial Efficacy of polyarginines. Data shows the average minimum inhibitory concentration (MIC) observed for each peptide ± SEM (n = 9) following an 18-h exposure of *Staphylococcus aureus* (*S. aureus*) and *Escherichia coli* (*E. coli*) to the oligoarginines.

To examine the pore-forming abilities of different structures of oligoarginines, carboxyfluorescein-containing liposomes were synthesised to simulate a phospholipid bilayer, where an increase in fluorescence intensity is expected to denote liposomal membrane disruption. The pore-forming abilities of the oligoarginine CPPs were initially tested on carboxyfluorescein-containing liposomes (Fig. 2A), where minimal penetration was observed from all tested oligoarginines. However, CPP R_9_ was the only oligoarginine length that recorded a significant increase in fluorescence intensity (p = 0.018) following the 10-min exposure of liposomes to the oligoarginines. Adding 0.1 % Triton X, acting as a positive control, caused lysis of liposomes. Consequently, significant leakage of fluorescein was detected as an increase in fluorescence intensity (p < 0.001), establishing the validity of the assay.

To further evaluate the membrane-disrupting capabilities of oligoarginine peptides, their antibacterial effects were examined against both gram-positive (*S. aureus*) and gram-negative (*E coli*) bacteria. The MIC values of oligoarginines against *E. coli* and *S. aureus* are shown in Fig. 2B. Across the tested oligoarginine concentration range (0.001–1 mM), neither R_3_ nor R_6_ displayed any reduction in bacterial growth against *S. aureus* over 18 h. However, R_9_ exhibited some degree of anti-bacterial activity, with an MIC of 1 mM, while R_12_ showed higher efficacy with an MIC of 0.61 ± 0.22 mM. A similar trend was observed with *E. coli*, where the shorter oligoarginines R_3_, R_6_, and R_9_ all showed no antibacterial efficacy over the 18-h duration. In contrast, R_12_ displayed the highest level of efficacy, recording an MIC of 0.94 ± 0.16 mM after the 18-h timeframe.

#### 3.1.1 CPPs cytotoxicity against mammalian cells

**Fig. 3:**
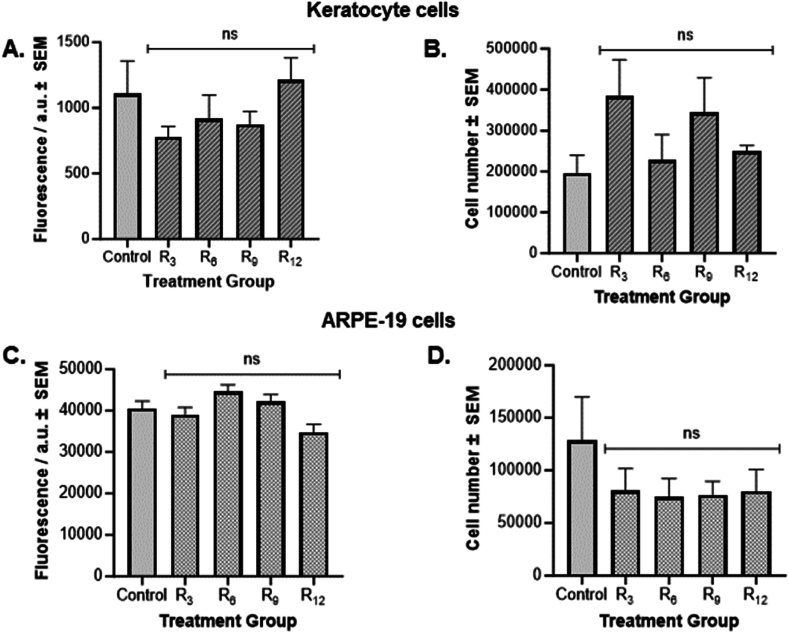
Cell toxicity of oligoarginine CPPs. *Metabolic rate of A) keratocyte and C) ARPE-19 cells following a 72-h exposure to* 56 μM *oligoarginine treatments using AlamarBlue® assay. Data shows the mean fluorescence intensity ± SEM (n = 9) of AlamarBlue*® *reagent. Fluorescence intensity is measured in arbitrary units (a.u.). Cell number of B) Keratocyte and D) ARPE-19 cells after oligoarginine treatment. Data shows the mean cell number ± SEM (n = 9). Significance was taken at p≤0.05 using One-way ANOVA and Dunnett's post-hoc test. ns denotes no significance when compared to Control.*

As the peptides demonstrated a toxic effect against bacteria, their potential toxicity against mammalian cells was examined. Both keratocyte and ARPE-19 cell monolayers were exposed to the oligoarginine peptides for 72 h. Cytotoxicity was evaluated by measuring the metabolic rate of cells using both the AlamarBlue® reagent and by performing a full cell count to provide a more comprehensive assessment of their toxicity profile *in vitro*. In both keratocyte and ARPE-19 cell monolayers, the oligoarginine CPPs recorded no significant difference in fluorescence intensity of AlamarBlue® when compared to PBS control cells (Fig. 3A–C). Similarly, exposure to CPPs did not result in significant differences in cell numbers compared to PBS control cells in either cell line (Fig. 3B–D). These results indicate that the CPPs were not cytotoxic to mammalian cells at the concentration tested.

#### 3.1.2 Cell penetration by CPPs

**Fig. 4:**
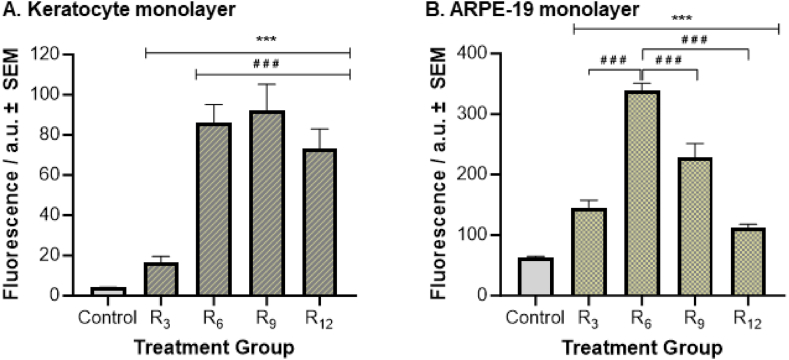
**Oligoarginine CPPs accessing the cell monolayer of A) Keratocyte and B) ARPE-19 cells. Control samples denote PBS only treatment.** Fluorescence intensity is measured in arbitrary units (a.u.). All data is presented as the mean ± SEM (n = 24). Significance was taken at p ≤ 0.05 using One-way ANOVA and Tukey's post-hoc test. ***p < 0.001 when compared to Control, ###p < 0.001 when A) compared to R_3_ and B) between each pairwise comparison.

To evaluate the penetration abilities of the peptides, keratocyte and ARPE-19 cells were treated with fluorescently-tagged oligoarginine CPPs (FAM-R_n_) and the cell monolayer absorbance was monitored for bound/internalised CPPs fluorescence (Fig. 4). A thorough washing procedure was employed to eliminate any remaining non-internalised or associated FAM-Rn. The level of oligoarginine present within cells in the monolayer was compared to that in untreated cells with no CPPs present (PBS control). In the keratocyte cell monolayers (Fig. 4A), the data showed that the level of CPPs fluorescence found within or associated with the cell monolayer was significantly higher for all peptides compared to PBS control (p < 0.001). The calculated uptake concentrations for FAM-R_3_, FAM-R_6_, FAM-R_9_, and FAM-R_12_ by keratocytes, as described in Section 2.8, were 8.0, 58.2, 62.7, and 48.9 nM, respectively. Additionally, cell monolayers treated with FAM-R_6_, FAM-R_9_, or FAM-R_12_ recorded a significantly higher level of fluorescence than the shorter peptide FAM-R_3_ (p < 0.001). However, no significant differences in fluorescence intensity between FAM-R_6_, FAM-R_9_, and FAM-R_12_ treated keratocyte cells were recorded.

Similarly, within ARPE-19 cell monolayers (Fig. 4B), cells treated with either length of oligoarginine presented statistically higher levels of fluorescence in comparison to the control cells (p < 0.001). An estimated 58.9 nM of FAM-R_3_, 197.6 nM of FAM-R_6_, 117.8 nM of FAM-R_9_, and 34.6 nM of FAM-R_12_ penetrated ARPE-19 cells. However, in contrast to keratocyte cells, FAM-R_6_-treated ARPE-19 cells displayed the highest level of fluorescence intensity, which was significantly different from the fluorescence levels observed in cells treated with FAM-R_3_, FAM-R_9_ and FAM-R_12_ (p ≤ 0.001). Furthermore, the addition of FAM-R_9_ resulted in a significantly higher level of fluorescence than both FAM-R_3_ and FAM-R_12_ (p ≤ 0.001), but no significant difference was observed between FAM-R_3_ and FAM-R_12_ (p > 0.066).

#### 3.1.3 CPPs effect on cell uptake of proteins

**Fig. 5:**
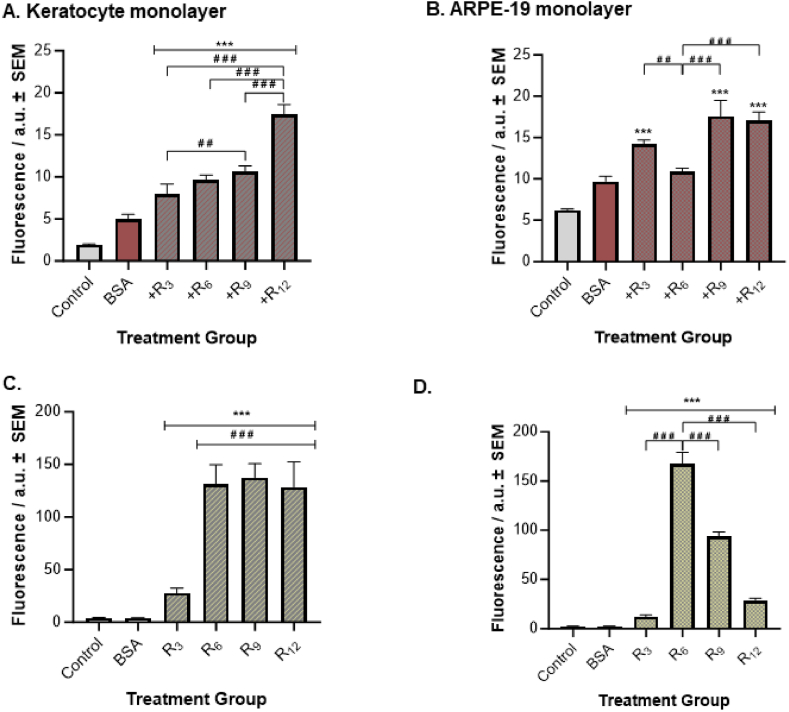
*BSA-Oligoarginine complex uptake. (A*–*B) BSA-TR fluorescence signal readings and (C*–*D) oligoarginine (FAM-R*_*n*_) fluorescence reading in Keratocyte and ARPE-19 cells. Control samples denote PBS-only treatment, and BSA samples denote BSA-TR-only control. Fluorescence intensity is measured in arbitrary units (a.u.). All data is presented as the mean ± SEM (n = 24). Significance was taken at p ≤ 0.05 using One-way ANOVA and Tukey's post-hoc test. ***p < 0.001 when compared to (A–*B) BSA control and (C*–*D) Control; ##p < 0.01 and ###p < 0.001 between each pairwise comparison.*

This study also examined the ability of different oligoarginine structures to carry proteins into the cell monolayers of keratocyte and ARPE-19 cells (Fig. 5). BSA-TR was used as a model cargo for proteins, and the cell absorbance was monitored for BSA-TR fluorescence to measure BSA cellular uptake. For both keratocyte and ARPE-19 monolayer experiments, a significantly higher fluorescence intensity value was observed for cells treated with BSA-TR-only compared to the PBS-only control cells (p ≤ 0.001). Thus, the BSA measurement served as a comparative control for the subsequent data analysis of BSA-TR delivery studies. In the keratocyte monolayers (Fig. 5A), a significantly higher fluorescence intensity for BSA-TR was recorded in cells treated with all FAM-R_n_/BSA-TR formulations compared to BSA control cells (p ≤ 0.001). Concentrations of 254.1, 311.5, 347.4, and 579.3 ng/mL of BSA-TR cellular uptake were estimated for FAM-R_3_, FAM-R_6_, and FAM-R_9_, and FAM-R_12_ containing formulations. Furthermore, an increasing trend between oligoarginine length and BSA-TR fluorescence intensity was observed, with chain length R_12_ recording the highest level of BSA-TR fluorescence. This intensity was significantly higher than FAM-R_3_, FAM-R_6_, and FAM-R_9_ containing formulations (p ≤ 0.001). Additionally, a statistically higher BSA-TR fluorescence level was recorded for FAM-R_9_/BSA-TR treated cells than FAM-R_3_/BSA-TR treated cells (p = 0.005).

In ARPE-19 monolayers (Fig. 5B), all FAM-R_n_/BSA-TR formulations led to a significant increase in BSA-TR fluorescence intensity compared to BSA control (p ≤ 0.001), except for FAM-R_6_/BSA-TR (p = 0.488). For FAM-R_3_/BSA-TR, FAM-R_6_/BSA-TR, and FAM-R_9_/BSA-TR, and FAM-R_12_/BSA-TR, approximately 91.8, 69.0, 115.1, and 111.5 ng/mL BSA-TR were taken up by ARPE-19 cells. Moreover, R_3_, R_9_, and R_12_ containing formulations led to significantly higher fluorescence levels for BSA-TR than the FAM-R_6_/BSA-TR formulation (p = 0.008, p ≤ 0.001, p ≤ 0.001, respectively). No statistically significant fluorescence differences were recorded for the remaining pairwise comparisons in either cell line.

We also examined whether the presence of a protein cargo in the formulation influences the cell-penetrating abilities of CPPs by measuring the FAM-R_n_ signal in both cell lines. For both keratocyte and ARPE-19 cell monolayers, a minimal fluorescence signal was detected for BSA-TR when using FAM-R_n_ absorbance settings, which was not significantly different from PBS-only control (p > 0.999), indicating that there was no crossover in fluorescence readings between oligoarginine (i.e. FAM-R_n_) and cargo (i.e. BSA-TR). In both keratocyte and ARPE-19 cells, the trend previously observed in the oligoarginine-only (FAM-R_n_) monolayers (Fig. 4) were similarly evident in the case of oligoarginine/cargo (FAM-R_n_/BSA-TR) formulations. In the keratocytes, all FAM-R_n_/BSA-TR formulations recorded a significantly higher FAM-R_n_ fluorescence intensity when compared to PBS control cells (p ≤ 0.001), with longer chain CPPs containing formulations displaying significantly higher fluorescence levels for FAM-R_n_ than the shorter peptide FAM-R_3_/BSA-TR formulation (p ≤ 0.001). Concentrations of 15.9, 90.9, 95.2, and 88.6 nM of FAM-R_3_, FAM-R_6_, and FAM-R_9_, and FAM-R_12_, respectively, were estimated to have penetrated keratocyte cell monolayers. In ARPE-19 cells, all FAM-R_n_/BSA-TR formulations recorded a significantly higher FAM-R_n_ fluorescence intensity when compared to PBS control cells (p ≤ 0.001), with R_6_ containing formulation displaying a significantly higher level of FAM-R_n_ fluorescence level than the other FAM-R_n_/BSA-TR formulations (p ≤ 0.001). The calculated uptake concentrations for FAM-R_3_, FAM-R_6_, and FAM-R_9_, and FAM-R_12_ by ARPE-19 cells were 5.9, 117.9, 64.6, and 17.7 nM, respectively.

#### 3.1.4 CPPs effect on cell uptake of small peptides

**Fig. 6:**
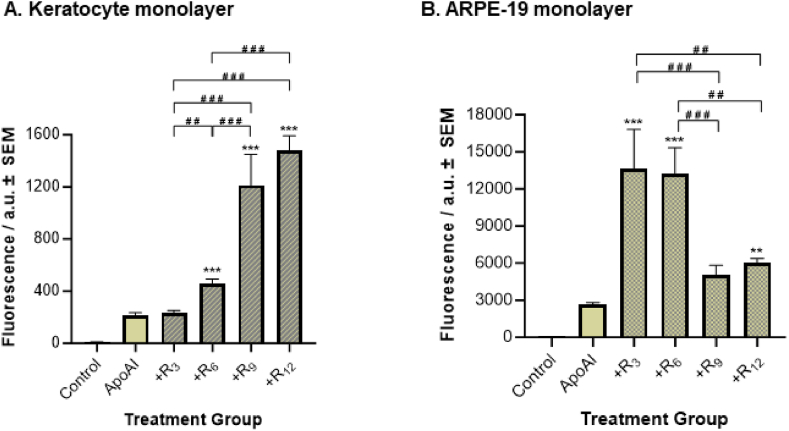
**ApoAI uptake by A) Keratocyte and B) ARPE-19 cells.** Control samples denote PBS-only treatment and ApoAI denotes FAM-ApoAI only treatment. Fluorescence intensity is measured in arbitrary units (a.u.). All data is presented as the mean ± SEM (n = 6). Significance was taken at p ≤ 0.05 using One-way ANOVA and Tukey's post-hoc test. **p < 0.01 and ***p < 0.001 when compared to ApoAI control, ##p < 0.01 and ###p < 0.001 between each pairwise comparison.

Just as proteins face challenges crossing cell membranes, small peptide-based therapies encounter the same limitations in drug delivery. Hence, we next investigated the ability of different oligoarginine CPPs to deliver small peptides into mammalian cells (Fig. 6). ApoAI acts as a model small peptide, and it was fluorescently tagged with FAM to allow its measurement. For all experiments, a significantly higher fluorescence intensity value was observed for cells treated with FAM-ApoAI only compared to the PBS-only control cells (p ≤ 0.001), and the FAM-ApoAI measurement was used as a comparative control for the following data analysis. In the keratocyte monolayers, only cells treated with the longer oligoarginines (i.e., R_6_, R_9_, R_12_) in ApoAI formulations recorded significantly higher levels of FAM-ApoAI fluorescence than ApoAI-only control cells (p ≤ 0.001). Additionally, the longest chains of R_9_ and R_12_ recorded significantly higher fluorescence levels than R_6_-containing formulations (p ≤ 0.001). No statistical difference in fluorescence intensity between R_9_/FAM-ApoAI and R_12_/FAM-ApoAI-treated cells was recorded (p = 0.109). In the ARPE-19 cells, only cells treated with R_3_-, R_6_-, and R_12_-containing formulations significantly increased FAM-ApoAI fluorescence levels more than FAM-ApoAI control cells (p ≤ 0.001). Unlike keratocyte cells, the shorter CPPs (i.e., R_3_ and R_6_) recorded the highest level of FAM-ApoAI fluorescence intensity compared to the longer CPP R_12_-containing formulation (p = 0.007 and p = 0.008, respectively).

## 4 Discussion

In this study, we investigated the pore-forming abilities of oligoarginine peptides by utilising a carboxyfluorescein-containing liposomes, with R_9_ being the only oligoarginine length causing a significant increase in fluorescence intensity. Additionally, the antibacterial efficacy of oligoarginine recorded differences depending on CPP length and bacterial class, with longer peptides exhibiting stronger antibacterial effects. Evaluation of mammalian cell toxicity showed no significant differences compared to control groups. Subsequent investigations on the membrane translocation abilities of oligoarginine in relevant *in vitro* cellular models relevant to ocular delivery revealed that both CPP translocation and cargo delivery across keratocyte and ARPE-19 cells were dependent on peptide length and cell type. Longer chains recorded a greater effect on cargo uptake in a corneal epithelium cell line (i.e. keratocyte cells), whereas shorter chains were more effective for cargo delivery in a retinal epithelium cell line (i.e. ARPE-19 cells).

Previous research investigating the role of oligoarginine peptides has identified multiple sequences that have proven effective in enhancing the penetration of therapeutic agents [16,17]. To comprehensively understand the behaviour of oligoarginine peptides, we initially examined their ability to create pores and penetrate a phospholipid bilayer liposome. This experimental approach allowed us to simulate the interactions between CPPs and the cell membrane. However, the oligoarginine peptides exhibited negligible penetration into the liposomal structures. Notably, only R_9_ showed an increase in fluorescence intensity of statistical significance compared to the control liposomes, suggesting some level of penetration through pore formation. This finding could suggest that pore formation might not be the primary mechanism responsible for cellular internalisation of the CPPs, with alternative processes such as endocytosis being their preferred mechanism [26].

The oligoarginines showed a stronger bacteriostatic effect on *S. aureus* than *E coli,* which agrees with *Sepahi* et al. (2017) [19]. This suggests that oligoarginine peptides may possess a stronger antibacterial effect against Gram-positive bacteria than Gram-negative bacteria. The species-related antimicrobial effect may be related to their different cell wall structures [27]. Additionally, in this study, we recorded a length-dependent antibacterial activity, as peptides with a larger number of arginine units, specifically R_9_ and R_12_, demonstrated greater antibacterial efficacy within an 18-h exposure period. This is in line with previous work carried out by Duncan et al. (2021) that demonstrated that the antifungal effect of oligoarginine peptides varies based on the length of the arginine chains, with an oligoarginine consisting of 11 or more arginine residues required for an antifungal effect to be observed [28]. Similarly, Guzman et al. (2013) indicated that oligoarginines with more arginine residues generally exhibited a more potent antimicrobial effect [29]. Nevertheless, it is possible that the shorter oligoarginines, R_3_ and R_6_, may require a longer exposure period to observe their antibacterial effects, as a study by Nikoi et al. (2018) demonstrated that the oligoarginine R_6_ displayed excellent antibacterial efficacy only after an extended incubation period of 15 days [13].

Importantly, although peptide length influenced their toxicity towards bacterial cells, none of the peptides exhibited cytotoxic effects on mammalian cells relevant to ocular drug delivery at a concentration of 56 μM. Our experiments involving keratocytes and ARPE-19 cells indicated that oligoarginines had no significant impact on either the metabolic rate or the cell number. These findings align with existing literature studies that have demonstrated the non-cytotoxic nature of oligoarginines such as R_6_ and R_7_ in other cell lines [9,30].

The ability of oligoarginine peptides to penetrate cells has been extensively documented in scientific literature [26,31,32]. Furthermore, studies involving oligoarginines coupled directly onto target compounds have revealed that the length of the arginine chain can influence cellular uptake [33]. However, to the best of our knowledge, the suitability of oligoarginines and the influence of chain length have not been specifically examined in cells relevant to ocular delivery despite the evident potential for this technology to enhance AMD clinical treatments significantly. Therefore, we evaluated the cell-penetrating abilities of oligoarginines in the presence and absence of a protein cargo in both human keratocyte (i.e. corneal epithelium component) and ARPE-19 (i.e. retinal blood barrier cellular model) cells [23,24]. Our study revealed that the cell-penetrating capabilities of oligoarginines depend on both the length of the peptide and the specific cell line. In both cell lines, all tested oligoarginine lengths (R_3_, R_6_, R_9_, and R_12_) demonstrated successful translocation into the cells at concentrations significantly higher than the control cells, regardless of the presence or absence of a protein cargo. However, in keratocyte cells, R_6_, R_9_, and R_12_ exhibited significantly greater translocation capabilities than R_3_, with no significant differences observed among the three longer chain lengths. This suggests that once a threshold level of six arginine residues is reached, penetration into the keratocyte cell is achieved, irrespective of further increases in peptide chain length. Therefore, no optimal oligoarginine length was identified for corneal epithelium penetration. This trend remained consistent even when the peptides were incubated with the protein cargo. On the other hand, in ARPE-19 cells, R_6_ displayed the highest level of cell penetration, significantly surpassing all other chain lengths, while R_3_ and R_12_ showed the lowest level of penetration. This pattern persisted regardless of the presence or absence of the protein cargo, indicating R_6_ may be the optimal length for effective penetration across the retinal–blood barrier.

Following the identification of trends in cell translocation of oligoarginines, their ability to deliver cargo was evaluated. To mimic drug delivery relevant to current AMD therapeutics (i.e., anti-VEGF agents), BSA was employed as a model protein cargo for anti-VEGF agents. In contrast, ApoAI, a novel candidate in AMD therapeutics, was used as a model for small peptide drugs [2,3]. Contrary to the observed trend in cell translocation of oligoarginines in the keratocyte cell line, a positive relationship between drug delivery and peptide length was indicated. Specifically, an increase in cellular uptake of both large and small molecule cargo was noted as the chain length of oligoarginine peptides increased, with R_12_ or both R_12_ and R_9_ demonstrating the highest level of BSA or ApoAI delivered to keratocyte cells, respectively.

Additionally, a contradicting trend to the peptide cell translocation was discerned in the capability of oligoarginines to deliver a protein cargo in the ARPE-19 cells. We identified R_6_ as the optimal oligoarginine chain length for achieving effective cell penetration across a cellular model of the retinal blood barrier. However, R_6_ exhibited the lowest level of BSA delivery into ARPE-19 cells, which was not significantly different from control cells. Therefore, the penetration abilities of oligoarginine might not translate to its drug delivery abilities. Nevertheless, in the context of small peptide cargo (i.e., ApoAI), the shorter oligoarginine lengths, R_3_ and R_6_, recorded the highest level of ApoAI delivery in ARPE19 cells. This contrasts with the findings in keratocyte cells, where peptides with more arginine residues (i.e., R_9_ and R_12_) showed superior cell delivery of ApoAI. The cell-dependent differences could indicate the interaction of oligoarginine with various transporters or other components representative of each cell line, as both the retinal epithelium and the corneal epithelium constitute a different protein expression profile [[34], [35], [36]].

Additionally, it could indicate the possibility of utilising chain length as a tool in the formulation process to selectively target specific regions of the eye. For instance, longer chain lengths may exhibit greater efficacy in delivering drugs to the anterior part of the eye, while shorter oligoarginines may be more suitable for the posterior region. Alternatively, a combination of shorter and longer oligoarginine variants may be the preferred approach to achieve a more uniform drug distribution throughout the eye.

Therefore, the penetration and drug delivery abilities of oligoarginines are dependent on the number of arginine units, cell type, and cargo type. However, it is important to note that the observed differences could also be influenced by the different concentrations used in these experiments, as the study by Tünnemann et al. (2008) suggests that the concentration of the peptide has an effect as well as the chain length [17]. As our study only evaluated the drug delivery potential of different lengths of oligoarginine CPPs in *in vitro* cellular models, future work should focus to corroborate these findings using *ex vivo* and *in vivo* models for ocular delivery, and additional mechanistic studies are required to further elucidate the interaction of oligoarginines with cell membranes and their components to provide better insights into their cellular translocation mechanisms.

## 5 Conclusion

This study provides insights into the intricate relationship between the structural characteristics of oligoarginine CPPs, their ability to penetrate mammalian cell types, and their potential to facilitate large and small-sized cargo delivery into cells, particularly in the context of topical ocular drug delivery. Our research proposes that the length and structure of oligoarginine could be used as a strategic tool in the formulation process to selectively target distinct eye regions. This includes using shorter chains for the posterior region of the eye, longer chains for the anterior segment, or a combination of both. Notably, pore formation did not appear to be the primary mechanism of oligoarginine-based delivery, with R_9_ being the sole peptide showing some degree of pore formation. In addition to oligoarginine drug delivery abilities, their antibacterial efficacy was contingent on the number of arginine residues and the specific bacterial species. It is worth emphasising that none of the evaluated oligoarginine structures exhibited toxicity towards mammalian cells relevant to the ocular barrier. This study extends our understanding of the applicability of oligoarginine CPPs as penetration enhancers for improving the delivery of therapeutics in an ocular topical formulation within the clinical context of AMD.

## Data availability

Data will be made available on request.

## CRediT authorship contribution statement

**Stefana Duca:** Writing – original draft, Methodology, Investigation, Formal analysis. **Naa Dei Nikoi:** Writing – review & editing, Methodology, Investigation. **Madeline Berrow:** Investigation. **Lois Barber:** Investigation. **Louise N. Slope:** Conceptualization. **Anna F.A. Peacock:** Writing – review & editing, Conceptualization. **Felicity de Cogan:** Writing – review & editing, Supervision, Methodology, Funding acquisition, Conceptualization.

## Declaration of competing interest

We have no conflicts of interest either financial or in any other way with regards to this work.
